# BMC Obesity reviewer acknowledgement 2014

**DOI:** 10.1186/s40608-015-0034-8

**Published:** 2015-04-03

**Authors:** Clare Partridge

**Affiliations:** BioMed Central, Floor 6, 236 Gray’s Inn Road, London, WC1X 8HB United Kingdom

## Abstract

The editors of BMC Obesity would like to thank all our reviewers who have contributed to the journal in Volume 1 (2014).

**Sean Adams**

USA

**Raghu Adya**

UK

**Carlos Alvarez-Dardet**

Spain

**Raghupathy Anchala**

India

**Shervin Assari**

USA

**Navoda Atapattu**

Sri Lanka

**Kathryn Backholer**

Australia

**Katherine Bauer**

USA

**Grzegorz Bazylak**

Poland

**Colin Bell**

Australia

**Rhonda Belue**

USA

**Farryl Bertmann**

USA

**Ken Blemings**

USA

**Susann Bluher**

Germany

**Maria Bryant**

UK

**Mansi Chopra**

India

**George Chrousos**

Greece

**Anna Ciao**

USA

**Eva Conceicao**

Portugal

**Molly Conroy**

USA

**Russell De Souza**

Canada

**Jeffrey Deiuliis**

USA

**Hélène Delisle**

Canada

**Flávio Demarco**

Brazil

**Jacopo Desiderio**

Italy

**Rosemary Duda**

USA

**Jozef Dulak**

Poland

**Mary Forhan**

Canada

**Rosanne Freak-Poli**

Australia

**Ismael Freitas**

Brazil

**Barry Garst**

USA

**Sanne Gerards**

Netherlands

**Folke Hammarqvist**

Sweden

**Thang Han**

UK

**Flo Harrison**

UK

**Lana Hebden**

Australia

**Setia Hermawati**

UK

**Natasha Howard**

Australia

**Terry Huang**

USA

**Melinda Hutchesson**

Australia

**Kevin Hwang**

USA

**Judy Jou**

USA

**Gregor Jurak**

Slovenia

**Masaharu Kagawa**

Japan

**Juliana Kain**

Chile

**Gregory Kaltsas**

Greece

**Rebecca Kanter**

USA

**Olga Khavjou**

USA

**Isurujith Kongalaliyanage**

Sri Lanka

**Bharati Kulkarni**

India

**Amy Luke**

USA

**Gian Mauro Manzoni**

Italy

**Anne Martin**

UK

**Katherine McCullough**

UK

**Daniel Mitchell**

UK

**Thomas Modéer**

Sweden

**Victor Mogre**

Ghana

**Paulina Nowicka**

Sweden

**Christine Olson**

USA

**Carsten Otto**

Germany

**Miranda Pallan**

UK

**Ashan Pathirana**

Sri Lanka

**Christine Peat**

USA

**Anna Peeters**

Australia

**Ralph Peterli**

Switzerland

**Antonio Prista**

Mozambique

**Kumari Rathnayake**

Sri Lanka

**Francisco Rios**

UK

**Anne Elizabeth Rogers**

UK

**Stefan Ryter**

USA

**Ian Sinha**

UK

**Amanda Staiano**

USA

**Steven Street**

Australia

**Michel Suter**

Switzerland

**Kurt Svardsudd**

Sweden

**George Tharakan**

UK

**Kimberly Truesdale**

USA

**Adam Tsai**

USA

**Indu Waidyatilaka**

Sri Lanka

**Carol Wham**

New Zealand

**Brandy Wicklow**

Canada

**Pujitha Wickramasinghe**

Sri Lanka

**Andrew James Williams**

UK

**Emmanuel Williams**

Ghana

**Jyh Eiin Wong**

Malaysia

**Lin Yang**

USA

**Ouliana Ziouzenkova**

USA

